# Effect of FSW Traverse Speed on Mechanical Properties of Copper Plate Joints

**DOI:** 10.3390/ma13081937

**Published:** 2020-04-20

**Authors:** Tomasz Machniewicz, Przemysław Nosal, Adam Korbel, Marek Hebda

**Affiliations:** 1 Faculty of Mechanical Engineering and Robotics, AGH University of Science and Technology, A. Mickiewicza Av. 30, 30-059 Krakow, Poland; machniew@agh.edu.pl (T.M.); pnosal@agh.edu.pl (P.N.); korbel@agh.edu.pl (A.K.); 2 Institute of Materials Engineering, Faculty of Materials Engineering and Physics, Cracow University of Technology, Warszawska 24, 31-155 Krakow, Poland

**Keywords:** FSW, copper, butt joint, mechanical properties, fatigue performance, traverse and rotation speed

## Abstract

The paper describes the influence of the friction stir welding travel speed on the mechanical properties of the butt joints of copper plates. The results of static and fatigue tests of the base material (Cu-ETP R220) and welded specimens produced at various travel speeds were compared, considering a loading applied both parallel and perpendicularly to the rolling direction of the plates. The mechanical properties of the FSW joints were evaluated with respect to parameters of plates’ material in the delivery state and after recrystallisation annealing. The strength parameters of friction stir welding joints were compared with the data on tungsten inert gas welded joints of copper plates available in the literature. The results of microhardness tests and fractographic analysis of tested joints are also presented. Based on the above test results, it was shown that although in the whole range of considered traverse speeds (from 40 to 80 mm/min), comparable properties were obtained for FSW copper joints in terms of their visual and microstructural evaluation, their static and especially fatigue parameters were different, most apparent in the nine-fold greater observed average fatigue life. The fatigue tests turned out to be more sensitive criteria for evaluation of the FSW joints’ qualities.

## 1. Introduction

Friction stir welding (FSW) is a relatively novel technique for joining materials, and thanks to the wide possibilities of application, it has been gaining popularity rapidly recent years [1]. In particular, this technique can be used as one of few methods for joining metals that are hard to weld or have physicochemical properties significantly different from each other, i.e., in cases when the use of conventional fusion welding is strongly limited and braze welding does not yield sufficient joint strength [2]. For this reason, FSW is commonly used not only for welding aluminium alloys [3,4] but also for many other metallic materials such as magnesium alloys [1,5,6,7], titanium alloys [1,8], steel [9,10], copper [11,12,13,14,15,16], and different combinations of dissimilar metals [1].

**Figure 1:**
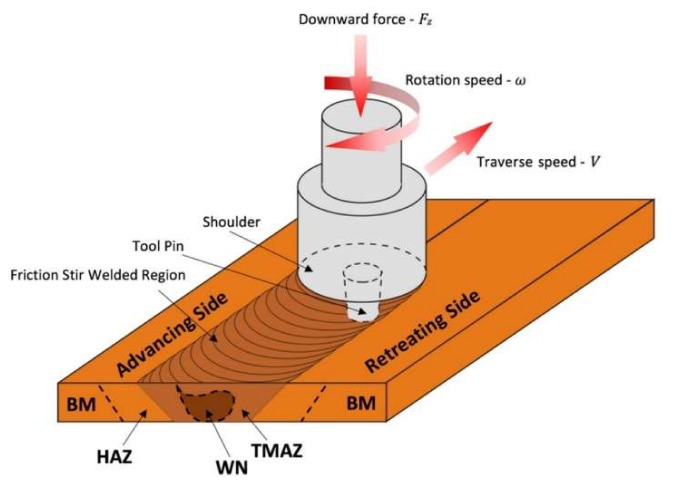
Schematic drawing of the friction stir welding (FSW) process.

Friction stir welding, as a solid-state process, is based on the plastic deformation of joined materials, elicited by using a special tool consisting in general of a shoulder and a mixing pin (Figure 1). The basic function of this tool is to generate heat, which enables the plasticisation of the materials, and then their mixing. Around the FSW joint, as shown in Figure 1, three characteristic zones within which the material properties have changed in relation to the base material (BM) are generally specified. These zones are: (i) the heat affected zone (HAZ), (ii) the thermo-mechanically affected zone (TMAZ) and (iii) the weld nugget (WN), where the material is fully recrystallized [1]. In addition, due to the complex motion of the working tool, there are two distinctive sides of the formed joint: the advancing side—where the tangential vector of the rotational speed of the tool is compatible with its travel speed vector, and the retreating side—where the senses of mentioned vectors are opposite (Figure 1).

The kinematics of the FSW process consist of two main motions, rotational and progressive, along the interface of joined materials. These motions correspond to the two basic parameters of the technological process which are the rotation speed (*ω*) and the traverse speed of the welding (*V*). These parameters affect the amount of heat generated during the process and thus the quality and properties of the wrought weld. Thus, for a given tool geometry, the mechanical properties of FSW joints are greatly influenced by the abovementioned technological parameters applied to the welding process as confirmed by numerous examples in literature [1,3,12,15,16]. A poor choice of these parameters may lead to various kinds of imperfections of the joints, significantly reducing their strength properties [17,18,19,20,21,22]. Although extensive research on the influence of FSW process parameters on the weld quality has already been conducted, existing publications most often concern aluminium alloys and static joint properties. The database on non-aluminium materials is more modest, and only rudimentary information can be found on the fatigue properties of produced joints. In particular, one of the materials that is still poorly known in this respect is copper, despite the fact that, due to the fact of its physical and chemical properties, copper combined with FSW technology is increasingly used in many areas of industry including the nuclear [22], energy [23] and automotive sectors [24]. Elements made in this way must have the required strength, not only in static but also in fatigue loading conditions, which can only be achieved through the appropriate choice of FSW process parameters.

A review of the literature provides divergent data on FSW parameters optimal for copper sheets. There are examples of significantly different combinations of rotation speed/traverse speed/plate thickness recommended for welding copper, e.g., 1000 rpm/30 mm·min^−1^/2 mm [11], 400 rpm/100 mm·min^−1^/3 mm [12], 250 rpm/61 mm·min^−1^/4 mm [13] and 1300 rpm/170 mm·min^−1^/6 mm [14]. Xue et al. [15], while welding copper plates with a thickness of 5 mm, applied various rotary speeds *ω* ≥ 400 rpm for the traverse speed of 50 mm/min and various traverse speeds *V* ≥ 50 mm/min for the rotary speed of 800 rpm. In the entire range of the considered parameters, they observed the systematic effect of joint strength increasing with increasing traverse speed for constant *ω* = 800 rpm, and with decreasing rotation speed at constant *V* = 50 mm/min. For the same sheet thickness, Khodaverdizadeh et al. [16] used both higher (*V* = 75 mm/min, *ω* = 600–900 rpm) and lower (*V* = 25 mm/min, *ω* = 600 rpm) process parameters relative to the research conducted by Xue et al. [15]. The obtained test results confirmed that the highest mechanical parameters of the joint were received for the combination of the lowest of the considered rotation speeds (600 rpm) and the highest traverse speed (75 mm/min). These results, however, are contrary to the generally expressed view that, due to the high thermal conductivity and high melting temperature of copper, a moderate welding speed is recommended, so that the right amount of heat energy can be generated [13]. On the other hand, excessive heat generation during welding of copper sheets hardened beforehand by cold rolling causes a more intensive reduction of strength parameters of the joint within the weld, due to the recrystallisation process. Because of the high degree of complexity of the processes occurring during FSW, it is important to determine the optimal technological parameters in order to obtain the highest possible strength properties of joints. For this purpose, the results of mechanical tests of butt joint specimens of electrolytic copper (99.998% Cu) with a thickness of *t* = 5 mm made by FSW with different traverse speeds are presented in this paper. Based on these, the influence of the applied process parameters on structural changes, microhardness and mechanical properties of the fabricated joints were analysed. When selecting optimal FSW traverse speed, static tests results are usually taken into account during research [11,12,13,14,15], but this work also considers the fatigue properties of FSW joints (i.e., fatigue lives). This is an approach rarely found in the literature but is considered by the authors as more sensitive criteria for evaluation of the FSW joints’ qualities. Moreover, data on the impact of FSW welding parameters on the properties of copper joints are usually limited to one specimen orientation. The results in this paper demonstrate that the variations of FSW parameters may have a qualitatively different effect on the properties of friction stir welds oriented longitudinally and transversely to the rolling direction of the jointed plates. After considering these additional criteria, the optimal combination of transverse and rotation speed selected in respect to the best strength parameters of FSW copper joints proved to be different from the proposals recommended so far in the literature.

## 2. Materials and Methods

**Figure 2:**
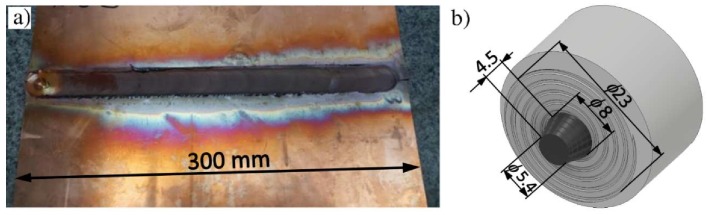
The FSW joint made at *ω* = 580 rpm, *V* = 60 mm/min (**a**) and geometry of the tool used for welding (**b**) (dimensions in mm).

**Figure 3:**
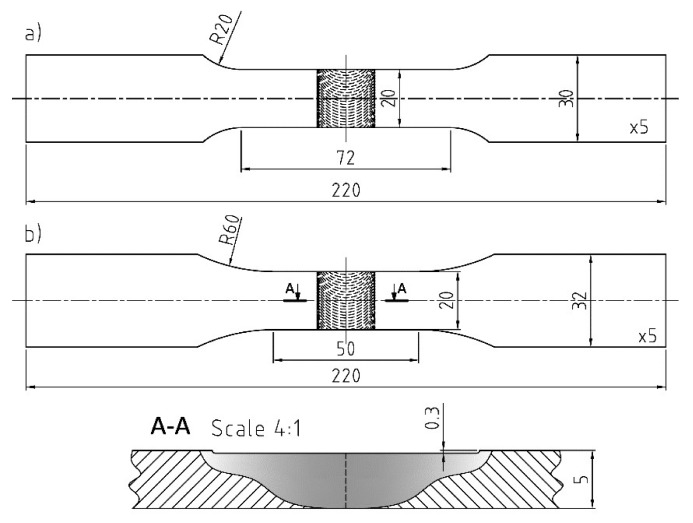
Specimen geometry used in static tests (**a**) and fatigue tests (**b**) (dimensions in mm).

Base material samples and FSW butt joints were fabricated from 5 mm thick plates of high purity electrolytic copper (Cu-ETP R220) with a chemical composition containing more than 99.9% Cu, according to the EN 573-1 standard. The FSW welds were performed on a conventional Jafo CNC milling machine at a constant rotation speed *ω* = 580 rpm and at three different traverse speeds: *V* = 40, 60 and 80 mm/min. The other FSW parameters were zero tilt angle, plunged depth of 0.3 mm, tool plunging speed of 2.5 mm/min and dwelling time of 5 s. The dimensions of the plate welded in such a way were approximately 300 mm × 300 mm. A view of the tool geometry used for welding and a representative example of the weld are presented in Figure 2. Samples from base material and welded plates for static and fatigue tests, with geometry shown in Figure 3, were cut out using the water-jet technique. The good quality (defect-free) of the FSW joints produced in accordance with the above parameters was confirmed by visual observation and ultrasonic evaluation.

**Figure 4:**
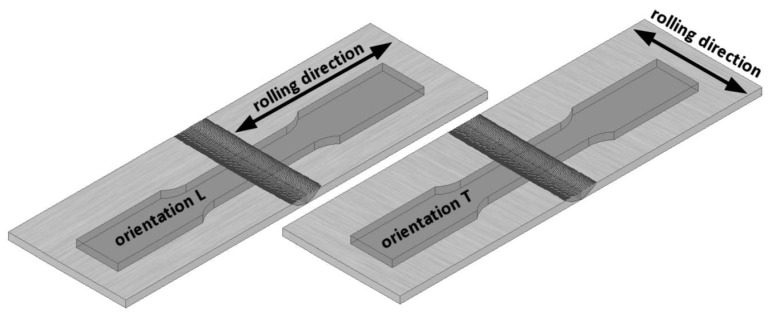
Schematic presentation of the longitudinal (L) and transversal (T) specimen orientation.

**Figure 5:**
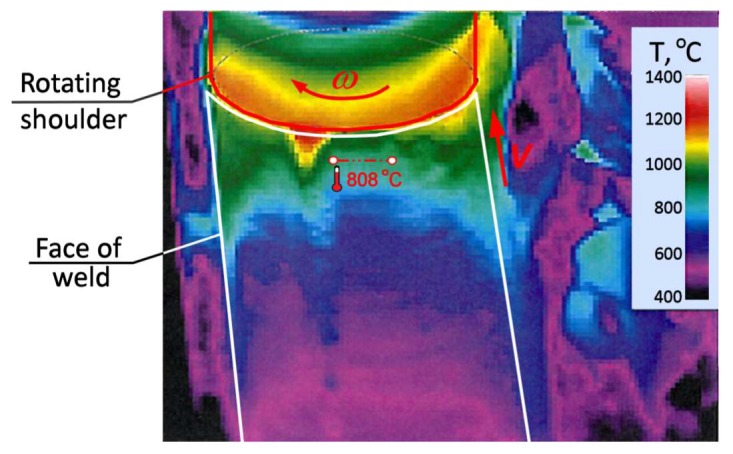
The temperature field (*T*) registered during FSW process by a thermal imaging camera on the copper plate surface behind the tool.

For FSW joints, the welds were located in the centre of the samples as presented in Figure 3. Specimens, both from the base material and from welded plates, were made for two sheet configurations, i.e., longitudinally (orientation L) and transversally (orientation T) to the direction of their rolling process, as presented schematically in Figure 4. The test plan was additionally extended by tensile tests of specimens subjected to earlier annealing at 600 °C. These results were considered as a reference point for the assessment of weld strength properties in the heat affected zone (HAZ). The annealing temperature was adopted based on temperature maps, as presented in Figure 5, registered by the thermal imaging camera on the surface of sheets during the welding process in the tool working area. Although the temporary weld temperature (*T*) just behind the rotating shoulder was about 800 °C, it stabilised a short distance from the tool at 600 °C.

Static and fatigue tests were carried out using the MTS 810 universal testing machine, with a load limit of 100 kN. Tensile tests were conducted according to the EN ISO 6892-1:2016 standard under displacement control at a rate of 0.5 mm/min. The strains were measured using axial extensometer (Epsilon 3542-025M-025-ST) with a gauge base of 25 mm and a measuring range of ±6.25 mm. The extensometer was mounted in the centre of the samples, covering the width of the weld when FSW joints were tested.

The stress-controlled constant-amplitude fatigue tests were performed with the stress ratio *R* = 0 using sinusoidal waveform loading at frequency *f* = 20 Hz. For the solid samples of the base material, four stress ranges were considered: Δ*S* = 210, 220, 230, 240 MPa. In the case of samples with an FSW joint, comparative fatigue tests were carried out only in the stress range of Δ*S* = 160 MPa due to the fact of their lower strength and large scatter of the results. The number of specimens subjected to monotonic tensile tests and fatigue tests is summarised in Table 1.

The microhardness measurements were performed for base material and three FSW joint specimens (i.e., one specimen per considered travel speed) in accordance with the ISO 6507-1 standard on Nexus 423A hardness equipment with a Vickers indenter, applying a loading force of 25 g and a measuring time of 10 s. The metallographic investigation was conducted using an Eclipse ME600 light optical microscope.

## 3. Results and Discussion

### 3.1. Microhardness Tests and Metallographic Analysis

**Figure 6:**
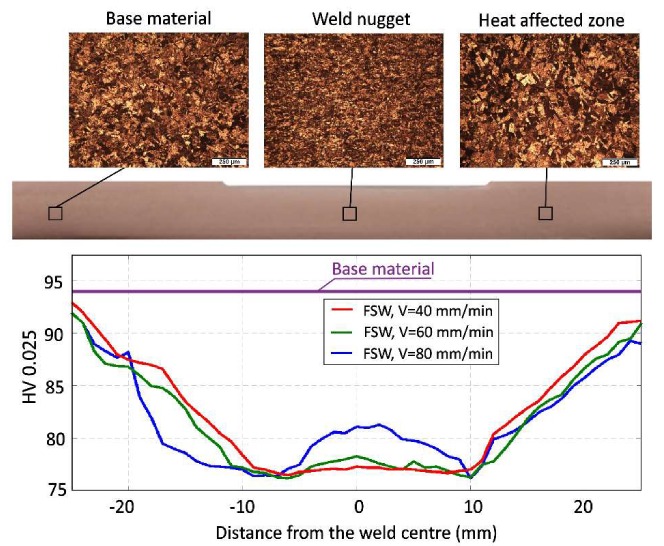
Microhardness profiles of the FSW joints corresponding to various traverse speeds and comparison of representative microstructure images in selected zones of the FSW copper joints produced with a traverse speed of 80 mm/min.

The microhardness distribution determined for the various traverse speeds on the cross-section of the joints along the centre of the specimens’ thickness is presented in Figure 6.

This figure also presents the representative microstructure images of the weld nugget, heat affected zone and base material for a joint welded at a speed of *V* = 80 mm/min. For this *V*-speed value, the profile of microhardness had a characteristic “W” shape (Figure 6) which is typical for most FSW joints [13,15,16,17,18,25]. For each of the traverse speeds used, a sudden decrease of microhardness in the HAZ was found. The minimum value of the microhardness (76.2 HV) was measured on the border of the HAZ and WN. The above trends corresponded well to the average grain sizes observed in the respective zones of the joint. Thus, the higher hardness in the WN zone than in HAZ was related to smaller grain size which was 10 μm for WN and 90 μm for HAZ. The base material, despite the larger grain size (approximately 70 μm), displayed a hardness and strength properties higher than those measured in the welded zone which is the effect of strain hardening arising from the sheet rolling process. A lower traverse speed value increases the amount of heat generated during the FSW process which has a direct impact on the lower hardness in the TMAZ and is manifested by a flatter microhardness profile for speeds of 60 and 40 mm/min (Figure 6). These trends, although widely shown in the literature [13,15,16,25], are contrary to other available data [26,27,28] showing that microhardness of the FSW copper joints increasing over the level related to the base material. For example, in the work of Berenji [26], for pure copper plates with the same thickness as those described in this paper (5 mm) and with very similar tool geometry and nearly the same rotation speed (600 rpm), a consistent increase of microhardness in the weld zone, over 75 HV for base material, was observed only when the traverse speed was above 25 mm/min. As in other works [27,28], this effect increased with the traverse speed. The highest reported ratios of such obtained microhardness of weld zone over the microhardness of the base material were (in HV): 90/75 [26], 114/77 [27] and 105/60 [28]. It may be noted that increased microhardness in the region of the FSW joints was observed in the case of soft copper conditions (microhardness below 80 HV) [26,27,28], while base material under higher strain with microhardness of over 80 HV results in a drop in hardness in the welded zone [13,15,16,25] which was also confirmed by the results of this paper.

### 3.2. Monotonic Tests Results

**Figure 7:**
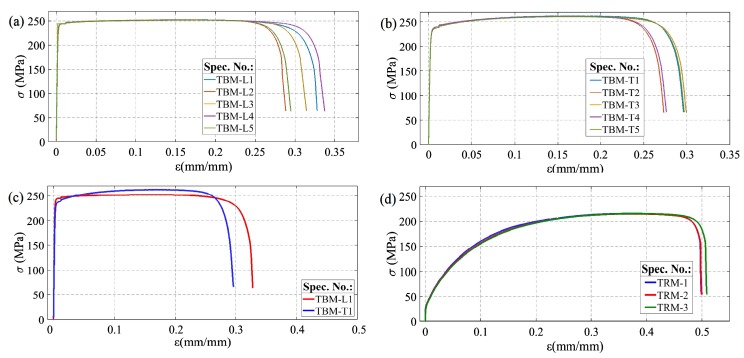
Tensile curves of solid samples: (**a**) base material—orientation L; (**b**) base material—orientation T, (**c**) base material—comparison of the representative curves for L and T orientation, (**d**) base material after annealing at 600 °C.

The tensile curves (*σ*—engineering stress; *ε*—engineering strain) for solid samples, including the base material specimens (specimens TBM), loaded parallel and perpendicularly to the rolling direction, and annealed specimens are presented in Figure 7. Determined from the static tensile tests, mechanical parameters of the solid and welded specimens, represented by yield stress (YS) ultimate stress (UTS) and reduction of area (AR), are summarised in Table 2.

As presented in Figure 7, the strength properties of solid sheets for the given orientation (L and T) were characterised by high repeatability, apart from a few percent spread of the strain at failure. Samples loaded along the rolling direction (TBM-L) above the elastic strain range showed almost perfectly plastic characteristics (Figure 7a). Comparison of the tensile curves for both orientations (Figure 7c) demonstrate that the strain at failure was slightly higher in the case of loading in accordance with the rolling direction of the sheet. In the transversal direction (specimen TBM-T, Figure 7c), where a slight strengthening effect was observed, the yield stress was lower by about 4% and the ultimate tensile strength was higher by about 3%, compared to longitudinal orientation (Table 2). The recrystalising annealing at 600 °C (specimens TRM, Figure 7d) caused a seven-fold decrease in yield stress and a decrease of about 17% in ultimate strength, with a simultaneous increase in strain at failure by close to 60%, and an increase in the value of reduction of area at failure by about 2%–4% (Table 2).

**Figure 8:**
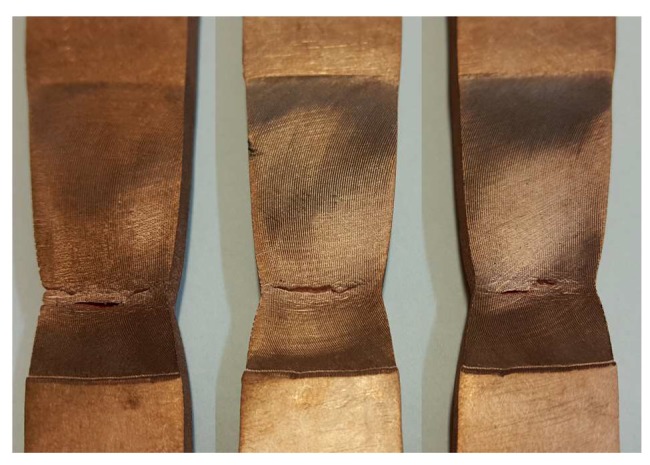
Fracture location of the tensile FSW joint samples.

**Figure 9:**
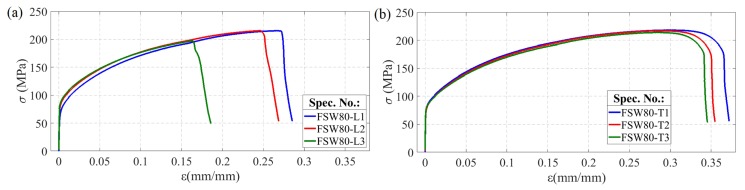
Tensile curves of FSW joint specimens produced with rotary speed *ω* = 580 rpm and traverse speed *V* = 80 mm/min: (**a**) orientation L; (**b**) orientation T.

**Figure 10:**
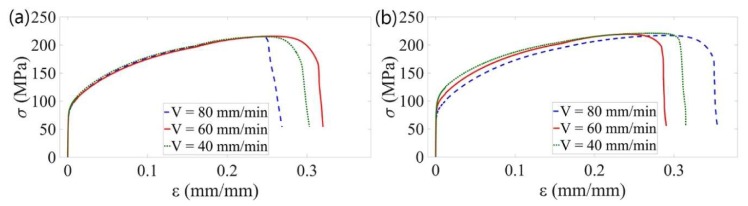
Comparison of the representative tensile curves of the specimens joined by FSW with various traverse speeds: (**a**) orientation L; (**b**) orientation T.

Monotonic tests of FSW joints comprised three tensile tests for each of the three traverse speeds considered. The cracking of the joints under static loading always occurred on the retreating side of the welds in the area of the TMAZ as shown in Figure 8. The exemplary tensile curves presented in Figure 9, corresponding to the highest traverse speed (i.e., *V* = 80 mm/min—FSW80 specimens), were characterised by the largest observed scatter, which was particularly pronounced for the longitudinal orientation (specimens FSW80-L, Figure 9a). Two samples from this series fractured before reaching the extreme point on the static tensile curve (Figure 9a), which was also manifested by lower values of strain at failure, as well as lower ultimate strengths (Table 2). The tensile curves representative of the joints welded at different traverse speeds are compared in Figure 10.

**Figure 11:**
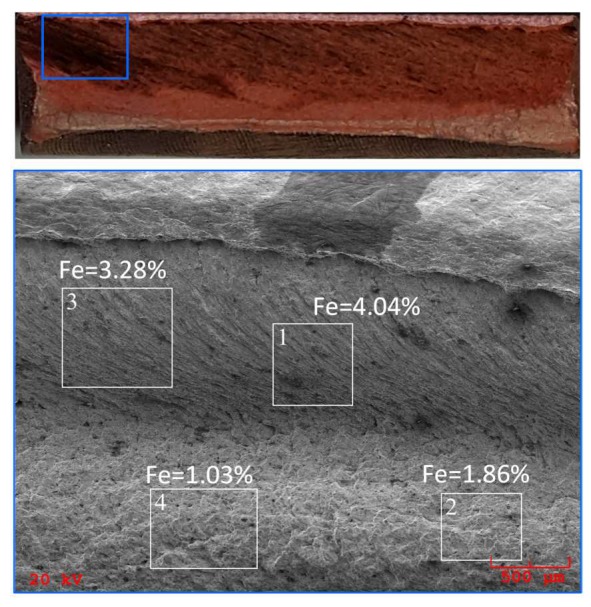
The fracture surface of the FSW80-L1 sample, showing areas of EDS analysis and iron content.

As may be observed, for longitudinal orientation the tensile curves regardless of *V*-speed are almost overlapped, except for the aforementioned effect of the lower ductility associated with the traverse speed of 80 mm/min (Figure 10a). In the transverse orientation (Figure 10b), a consistent trend of decrease of YS and UTS values with increasing traverse speed was found, which is qualitatively different to the available literature data [15,16,29]. Assuming that reduction of the joint strength parameters is induced by a copper recrystallisation process in the HAZ, this effect should be more intensive for a lower *V*-value, which corresponds to the higher amount of thermal energy generated during the FSW process. Besides the aforementioned experimental data, this mechanism is also confirmed by numerical simulations of the FSW welding process [30]. Macroscopic analysis of tensile fracture surfaces of FSW joints did not show distinct defects in the weld structure. The only dark streaks were observed on the fracture of the FSW specimen with the lowest strength parameters (sample FSW80-L1) as presented in Figure 11. Energy-dispersive spectroscopy (EDS) analysis of the areas marked on Figure 11 revealed the presence of iron, probably from the FSW tool, with content ranging from 1% to 4%.

**Figure 12:**
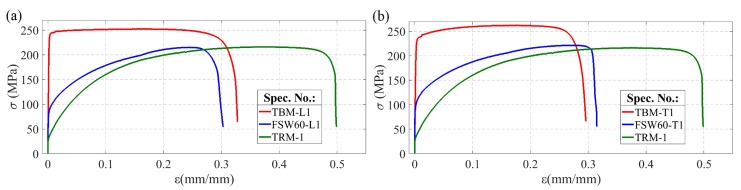
Comparison of representative tensile curves of base material (TBM), annealed material (TRM) and FSW specimens welded with *V* = 60 mm/min (FSW60): (**a**) orientation L; (**b**) orientation T.

Reduction of the strength properties of FSW joints in relation to solid material samples, as the effect of the copper recrystallisation in the heat affected zone, is clearly visible in Figure 12.

**Figure 13:**
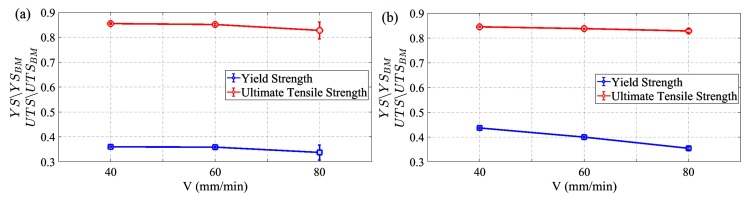
The mechanical parameters of FSW copper joints (YS and UTS) at different traverse speeds, normalised by base material properties (YS_BM_ and UTS_BM_): (**a**) longitudinal orientation, (**b**) transversal orientation.

The ratios of the mechanical parameters observed for FSW joints at different traverse speeds to the parameters specific to the solid material (YS/YS_BM_ and UTS/UTS_BM_) for both sample orientations are shown in Figure 13. Resulting from the FSW process, the YS decreased on average by more than 60% (Figure 13), i.e., from 230–240 MPa to 80–100 MPa (Table 2). This effect was almost independent of the rolling direction of the sheet. A smaller but still significant decrease, amounting to 15%, was also observed for UTS. Figure 13 reveals a consistent trend of decreasing strength parameters of FSW joints with increasing traverse speed. Although (with the exception of the YS changes for transversal orientation (Figure 13b)) the variations presented in Figure 13 are very moderate, these trends are, in qualitative terms, opposite to those described in the literature [15,16,27]. In general, with increasing traverse speed, the amount of thermal energy generated during FSW decreases [4]. This contributes to maintaining higher strength parameters due to the lower intensity of the copper recrystallisation process. The excessive scatter of the strength parameters observed in Figure 13a (longitudinal orientation) for the speed *V* = 80 mm/min confirms the earlier mentioned objections regarding this series of joints.

Nevertheless, the strength parameters of FSW welds, particularly the YS, remain higher in relation to the properties of the copper subjected to recrystalising annealing (Figure 12, Table 2). In addition, these FSW joint parameters were also significantly higher—UTS by close to 30%, and YS by about 67%—than the literature values for properties of butt joints welded from pure copper sheets using the tungsten inert gas (TIG) method [31]. This may be due to the strong plastic deformation of the material caused by the tool acting on the plates being joined by the FSW process, which does not occur in fusion welding (including TIG welding). The result is a refined microstructure formed in the region of the weld nugget and in the zone of thermo-mechanical impact (see Section 3.1.) which improves the mechanical properties of the joints. In addition, the temperature of the FSW process is lower than at the fusion welding, thus avoiding problems of porosity, cracking, sheet distortion and large size of heat affected zone. The higher strength of FSW joints compared to solid copper samples after annealing indicates that the reduction of mechanical properties of these joints is predominantly related to the loss of the cold-work hardening effect as a result of the recrystallisation process in the heat affected zone of the joint. Therefore, the improvement of strength parameters of the joints made using the FSW technique from strain-hardened copper sheets is possible by limiting the material’s heating during welding. This, in addition to selecting the optimal FSW parameters, may be implemented using a cooling medium during the FSW process [29].

### 3.3. Fatigue Test Results

**Figure 14:**
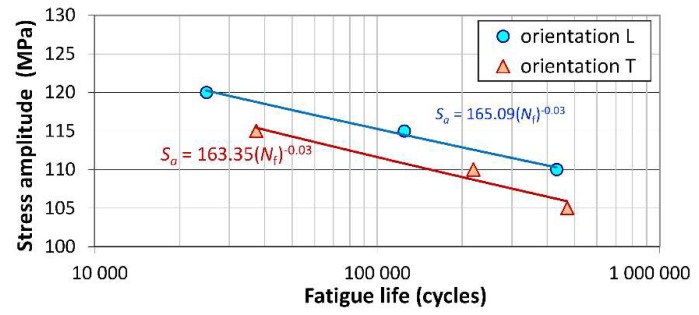
*S*–*N* curves of Cu-ETP R220 base material specimens.

The fatigue test results for base material specimens for both orientations are compared in the form of *S*–*N* curves in Figure 14. As may be observed, the specimens loaded perpendicularly to the rolling direction of the sheet repeatedly showed slightly lower fatigue strength (of about 4%) than the samples of longitudinal orientation. This is opposite to the trend of ultimate strength changing for both orientations under monotonic loading (Table 2).

**Figure 15:**
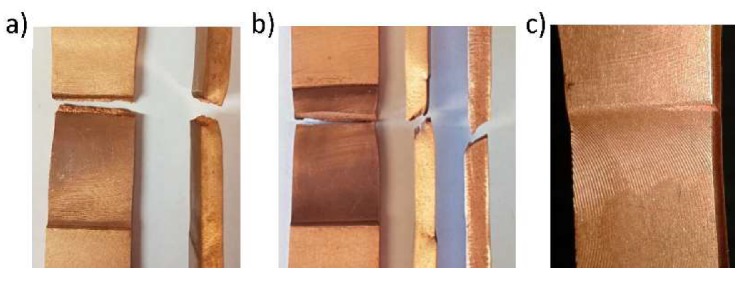
Location of the fatigue cracks observed in FSW joints: (**a**) crack at the edge of the weld, (**b**) crack at some distance from the edge of the weld, (**c**) typical mode of fatigue crack initiation.

In all specimens with T-orientation, and in specimens with L-orientation welded at a speed of *V* = 60 mm/min, fatigue cracks occurred on the retreating side in the plane of the notch formed along the edge of the rotating tool plunge as illustrated in Figure 15a. In the samples with longitudinal orientation welded at speeds of *V* = 40 mm/min and 80 mm/min, fatigue cracks were also located on the retreating side but, similar to static tests, at some distance from the weld edge (Figure 15b). The plane of cracks developed at the welds’ edges was nearly perpendicular to the face of the sample (Figure 15a), while in the TMAZ (Figure 15b) the cracks propagated on a plane inclined to the face of sample at an angle of about 45–60 degrees. In general, the cracks initiated in the corner of the sample on the face side of weld as illustrated in Figure 15c.

**Figure 16:**
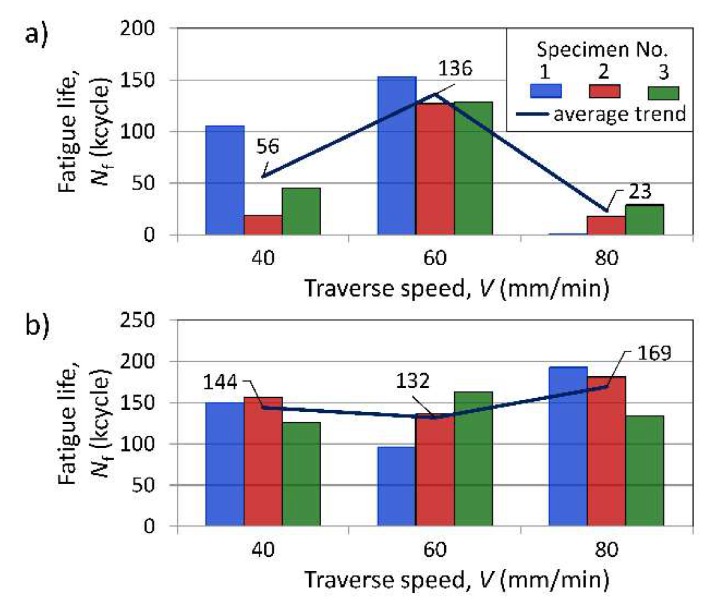
Influence of traverse welding speed on fatigue life of FSW joint specimens (*R* = 0, Δ*S* = 160 MPa): (**a**) orientation L; (**b**) orientation T.

Fatigue strength of the specimens containing FSW joints was significantly lower compared to base material specimens. In the tests of the FSW joint specimens with longitudinal orientation (Figure 16a), the highest fatigue lives (*N*_f_), and simultaneously the most repetitive results were obtained for samples welded at a speed of *V* = 60 mm/min. For this configuration, other considered traverse speeds, i.e., the lower (*V* = 40 mm/min) and the higher (*V* = 80 mm/min), led to significant lower fatigue lives and much greater scatter of the test results. One of the specimens welded with a traverse speed of 80 mm/min cracked during the first loading sequence. For all samples from the two series, of the lowest fatigue life was characterised by the distinct location and morphology of the cracks. FSW joint specimens oriented transversally to the rolling direction (Figure 16b) were characterised by more repeatable results, regardless of the traverse speed used. For this orientation, the difference between the average fatigue lives for specimen series associated with all considered *V*-values did not exceed 22%. In contrast to samples with L orientation, the highest fatigue lives were observed for the joint specimens welded at a speed of *V* = 80 mm/min. Concurrently, the speed of *V* = 60 mm/min, which had given the highest durability for the samples with L-orientation, provided the lowest fatigue lives in the case of transversally oriented specimens.

Comparing the fatigue test results obtained for specimens cut parallel (L) and perpendicularly (T) to the rolling direction, it may be pointed out that the traverse speed *V* = 60 mm/min was the most advantageous of the parameters used, due to the highest level of repeatability of observed fatigue lives. For this *V*-value, the difference in average fatigue lives for both specimen orientations was only 3%, while for the speed of 40 mm/min the average fatigue life for the transversal orientation was more than three times higher, and for a speed of 80 mm/min it was more than ten times higher, compared to the longitudinal orientation of the joints. Even for the most favourable traverse speed, the fatigue strength of specimens containing FSW joints, at fatigue life of 130,000 cycles, was lower by about 30% compared to the base material specimens.

Available research papers concerning the impact of FSW parameters on the mechanical properties of copper FSW joints [11,12,13,14,15,16,25,26,27,28,29] usually focus on the microstructure, hardness profiles and static properties of joints. To the best knowledge of the authors, there are no publications on FSW copper joint properties which reference various joint orientation and/or fatigue properties. However, as the results of this paper demonstrate, accounting for the last factors may qualitatively change the conclusions about the strength properties of considered joints. As follows from Section 3.2. (Table 2 and Figure 13), the traverse speed had a rather moderate effect on the static strength parameters of the tested FSW joints. For tested FSW copper joints, contrary to available literature data [15,16,27], YS- and UTS-values decreased as traverse speed increased. However, this trend was significant only for YS-variation in the case of joints with T-orientations, for which the YS decreased by almost 19% with an increase of *V*-speed from 40 to 80 mm/min. For the other cases, the changes in YS and UTS values did not exceed 6%. Taking into consideration only the tensile and microhardness properties of joints, the traverse speed of 40 mm/min could be selected as the optimal one. Fatigue tests, however, proved to be more sensitive criteria for evaluation of the FSW joints’ qualities. These tests clearly confirmed the weakness of the joints made at a speed of 80 mm/min with longitudinal orientation (which was earlier indicated only by the scatter of tensile test results (Figure 13)) and revealed the poor quality of the joints produced at a speed of 40 mm/min for the same orientation which had not been demonstrated earlier by other studies.

## 4. Conclusions

In this work, the properties of pure copper FSW joints were studied by means of microhardness, tensile and fatigue tests as well as microstructure and fractography analysis. As the main variables in conducted tests, three values of traverse speed and two different plate orientations in terms of rolling direction were considered. The constant rotation speed value (580 rpm) and different traverse speeds (40, 60 and 80 mm/min) were preliminarily selected for producing defect-free joints in terms of their visual and ultrasonic evaluations. The most relevant conclusions can be summarised as follows:

1. In the area of tested FSW joints, a significant decrease in microhardness was observed relative to the base material property. The microhardness profiles determined for the cross-section of the joints had a W-type shape with a sudden decrease in microhardness in the HAZ and a tendency towards a slight increase in WN which, in the qualitative sense, correlated well with the average grain sizes observed in these zones. However, the data available in the literature also indicate that opposite trends of microhardness changes are possible. Summarising the obtained results and the literature data, it may be generalised that increase of the microhardness in the region of FSW joints is observed for softer copper (microhardness below 80 HV), while the drop of hardness in the FSW zone is typical for copper with a higher degree of strain hardening.
2. Tensile strength parameters of tested FSW joints were significantly lower than those of base material properties which was predominantly related to the loss of the cold-work hardening effect as a result of the recrystallisation process in the HAZ. Resulting from the FSW process, the yield stress decreased on average by more than 60%, and ultimate stress fell by about 15%. For each of considered traverse speed, the FSW joints showed considerably higher static strength properties than similar joints made by TIG welding. These properties were also higher than those of the base material after annealing. Regardless of traverse speed, the tensile strength parameters of FSW joints were higher for transversal than for longitudinal orientation. Tensile tests revealed a consistent trend of decreasing strength parameters of FSW joints (i.e., yield stress and ultimate stress) with increased traverse speed. Although this effect is moderate, it is qualitatively different from the data reported so far in the literature.
3. The fatigue tests turned out to be more sensitive criteria for evaluation of the FSW joints’ qualities compared to other kind of examinations applied in this work. These tests clearly confirmed the weakness of the FSW joints produced at a speed of 80 mm/min in longitudinal orientation, which was earlier indicated only by the scatter of tensile test results, and revealed the poor quality of the joints made at a speed of 40 mm/min for the same orientation, which had not been predicted earlier by other studies. All samples from the two series of the lowest fatigue life were characterised by distinct location and morphology of the cracks. Considering all static and fatigue tests—for given plates, adopted tool geometry and specified rotary speed—the traverse speed of 60 mm/min proved to be the most advantageous. It should be noted that conclusions on the quality of the FSW joints resulting from fatigue tests would be different if they were referred separately to each of the considered joint orientations. This means that the variations of FSW parameters may have a qualitatively different effect on the properties of friction stir welds oriented longitudinally and transversely to the rolling direction of the joined plates.

## Figures and Tables

**Table 1:** Tensile and fatigue test matrix.

Kind of Test:		Tensile		Fatigue	
Specimen Orientation:		Longitudinal	Transversal	Longitudinal	Transversal
Specimen type	Base Cu-ETP R220	5	5	3	3
	FSW: *V* = 80 mm/min	3	3	3	3
	FSW: *V* = 60 mm/min	3	3	3	3
	FSW: *V* = 40 mm/min	3	3	3	3
	Annealed Cu-ETP	3	-	-	-

**Table 2:** Mechanical properties of tested samples.

Sample Type	Longitudinal Orientation (L)			Transversal Orientation (T)		
	YS (MPa)	UTS (MPa)	AR (%)	YS (MPa)	UTS (MPa)	AR (%)
Base Cu-ETP R220	242 ± 1.5	252.7 ± 1.5	67.3 ± 6.7	232.0 ± 0.7	261.2 ± 0.4	69.2 ± 1.0
FSW: *V* = 40 mm/min	87.7 ± 0.5	216.0 ± 0.8	69.4 ± 3.5	101.3 ± 0.5	220.7 ± 0.5	53.1 ± 7.2
FSW: *V* = 60 mm/min	87.3 ± 0.5	215.0 ± 0.8	70.5 ± 1.5	92.7 ± 0.5	218.7 ± 0.5	60.1 ± 4.2
FSW: *V* = 80 mm/min	82.0 ± 7.3	209.0 ± 8.5	40.3 ± 2.9	82.3 ± 0.9	216.3 ± 1.7	61.2 ± 2.2
Annealed Cu-ETP	33.0 ± 0.8	215.7 ± 0.5	71.3 ± 1.9	-	-	-
TIG welded joint [16]	53	168	5.7	-	-	-
